# The use of Diffusion Kurtosis Imaging for the Differential Diagnosis of Alzheimer’s Disease Spectrum

**DOI:** 10.3390/brainsci13040595

**Published:** 2023-03-31

**Authors:** Huiqin Zhang, Zuojun Wang, Koon-Ho Chan, Yat-Fung Shea, Chi-Yan Lee, Patrick Ka-Chun Chiu, Peng Cao, Henry Ka-Fung Mak

**Affiliations:** 1 Department of Diagnostic Radiology, National Cancer Center/National Clinical Research Center for Cancer/Cancer Hospital, Chinese Academy of Medical Sciences and Peking Union Medical College, Beijing 100021, China; 2 Department of Diagnostic Radiology, Li Ka Shing Faculty of Medicine, The University of Hong Kong, Hong Kong 999077, Chinamakkf@hku.hk (H.K.-F.M.); 3 Department of Medicine, School of Clinical Medicine, Li Ka Shing Faculty of Medicine, The University of Hong Kong, Hong Kong 999077, China; 4 Alzheimer’s Disease Research Network, The University of Hong Kong, Hong Kong 999077, China; 5 Division of Geriatrics, Queen Mary Hospital, Hong Kong 999077, China; 6 State Key Laboratory of Brain and Cognitive Sciences, The University of Hong Kong, Hong Kong, 999077, China

**Keywords:** Alzheimer’s disease, diffusion kurtosis imaging, structural imaging, hippocampus, diagnosis, mean diffusivity, mean kurtosis

## Abstract

Structural and diffusion kurtosis imaging (DKI) can be used to assess hippocampal macrostructural and microstructural alterations respectively, in Alzheimer’s disease (AD) spectrum, spanning from subjective cognitive decline (SCD) to mild cognitive impairment (MCI) and AD. In this study, we explored the diagnostic performance of structural imaging and DKI of the hippocampus in the AD spectrum. Eleven SCD, thirty-seven MCI, sixteen AD, and nineteen age- and sex-matched normal controls (NCs) were included. Bilateral hippocampal volume, mean diffusivity (MD), and mean kurtosis (MK) were obtained. We detected that in AD vs. NCs, the right hippocampal volume showed the most prominent AUC value (AUC = 0.977); in MCI vs. NCs, the right hippocampal MD was the most sensitive discriminator (AUC = 0.819); in SCD vs. NCs, the left hippocampal MK was the most sensitive biomarker (AUC = 0.775). These findings suggest that, in the predementia stage (SCD and MCI), hippocampal microstructural changes are predominant, and the best discriminators are microstructural measurements (left hippocampal MK for SCD and right hippocampal MD for MCI); while in the dementia stage (AD), hippocampal macrostructural alterations are superior, and the best indicator is the macrostructural index (right hippocampal volume).

## 1. Introduction

Alzheimer’s disease (AD), with a spectrum spanning from preclinical subjective cognitive decline (SCD) to prodromal mild cognitive impairment (MCI) and AD, is the leading cause of dementia, a global healthcare challenge to patients and the elderly population [1]. Therefore, differential diagnosis of the AD spectrum is vital for disease management, especially at the early stage. In past decades, the diagnostic criteria for AD have been changed several times while increasingly incorporating the radiological, molecular, and anatomical/microstructural imaging modalities [2,3,4,5,6,7]. In the 1980s, AD diagnosis primarily depended on the core clinical signs and symptoms, such as memory decline and cognitive impairment [2]. In 2007, brain atrophy on magnetic resonance imaging (MRI) was introduced into the international working group (IWG) criteria of AD diagnosis [3]. In addition, molecular positron emission tomography (PET) imaging tracers, such as amyloid and tau biomarkers, were added to the diagnostic criteria, which might be a more direct way of revealing the pathology of AD [4,5,7]. Although AD diagnostic criteria have been improved, accurate AD staging for patients with early signs of cognitive symptoms remains challenging [7].

The hippocampus plays a central role in human cognition. Mounting evidence shows hippocampal atrophy, the most sensitive imaging feature for AD in the late stage [8,9]. For example, a previous study identified bilateral hippocampal atrophy as an indication of AD using MRI [10]. Apart from the anatomical changes resulting from neural density loss in the later stage of AD, diffusion tensor and kurtosis imaging (DTI and DKI) can assess the microstructural abnormalities of the pathological brain before the volume reduction [11]. The commonly used measurements of DTI and DKI are fractional anisotropy (FA), mean diffusivity (MD), axial diffusivity (AxD), radial diffusivity (RaD), or mean kurtosis (MK) [12], which could be effective in assessing microstructural and neurological changes associated with AD and cognitive impairment [13]. For example, Hanne Struyfs et al. compared the DKI parameters of white matter (WM) among normal controls (NCs), MCI, and AD, and their results suggested that MD and MK of the splenium of the corpus callosum and the MD of the inferior longitudinal fasciculus may be regarded as indicators for AD [14]. In addition, Li et al. reported bilateral hippocampal MK changes in AD [15]. However, it is unclear which biomarker is more effective in differentially diagnosing the AD spectrum, including SCD, MCI, and AD, i.e., the macrostructural alterations (volume reduction) or microstructural changes (diffusion tensor and kurtosis changes from DTI and DKI) of the hippocampus. 

The predementia stages for AD include preclinical SCD and prodromal MCI [16,17]. Differentiating SCD and MCI is crucial for early intervention, which could slow down the progression of dementia [18]. The differentiation between MCI and AD is especially necessary, because earlier intervention with disease-modifying treatment, e.g., lecanumab, in the MCI stage may be beneficial [19,20]. Previous DKI experiments only studied the brain imaging features in MCI and AD cohorts [21,22,23,24], while the DKI characterization of the whole AD spectrum has been unsolved thus far. In addition, most of the published DKI studies have been focused on WM [25,26], and only a few studies explored grey matter (GM), such as the hippocampus. SCD belongs to the preclinical stage, but no quantitative imaging marker has been developed.

Therefore, in this study, we characterized the anatomical MRI and DKI in four age-matched groups: NCs, SCD, MCI, and AD. Specifically, the objectives of this study were (1) to compare the differences in volume reduction and DKI parameters of bilateral hippocampus among NCs, SCD, MCI, and AD; (2) to compare the differential diagnostic value of volume loss and DKI measurements of bilateral hippocampus among SCD, MCI, and AD; and (3) to determine whether DKI can detect AD at an early stage.

## 2. Materials and Methods

### 2.1. Participants

The local institutional review board approved this study. The informed consent forms were acquired from the cognitively preserved participants or the caregivers for the demented patients. All subjects (above 55 years of age) were prospectively enrolled in the study via the University of Hong Kong clinic between June 2017 and June 2019. All of them were right-handed. Participants with other neurological conditions (such as stroke, tumors, infection, movement disorders, demyelinating diseases, seizure, and migraine) and psychiatric disorders were excluded. Detailed descriptions of inclusion and exclusion criteria for the participants have been reported previously [27,28,29]. AD and MCI were defined mainly according to the core criteria of AD and MCI from the National Institute on Aging-Alzheimer’s Association separately [4,17]. SCD was determined based on recommendations by the IWG [30]. The final diagnosis was made by a panel consisting of one neuroradiologist and two geriatricians based on clinical manifestations, neuropsychological features, amyloid PET findings (a positive amyloid finding on PET was also required for a definitive diagnosis of AD), and anatomical MRI data. Finally, 64 participants were included (11 SCD, 37 MCI, and 16 AD), and another 19 elderly normal controls (NCs) were also referred. All the normal control subjects were age- and sex-matched with those of the disease groups. The NC should not have any disorders or any psychological or psychiatric conditions. However, information such as educational level, social status, and depression level was unavailable for some subjects. Therefore, we didn’t set these factors as covariances in the group analysis. These factors might affect a few categories in cognitive assessment but were secondary and weakly associated with mental symptoms; thereby, they were negligible [31].

All the participants completed the MRI scanning, while only the patients underwent the amyloid PET scanning. The clinical features were evaluated for each subject. In addition, the comprehensive cognitive test was assessed using the Hong Kong version of the Montreal Cognitive Assessment (HK-MoCA) [32]. 

### 2.2. MRI Acquisition

The MRI scanning was performed on a 3T scanner (Achieva, Philips Healthcare, Best, the Netherlands) with 32-channel head coils for signal receptions. The MRI protocol included three-dimensional (3D), T1-weighted magnetization prepared rapid gradient echo (MPRAGE) (repetition time (TR) = 6.8 ms, echo time (TE) = 3.2 ms, inversion time (TI) = 900 ms, matrix = 256 × 256, field of view (FOV) = 240 × 256 × 204 mm^3^, slice thickness = 1.2 mm), 3D T2-weighted fluid-attenuated inversion recovery (FLAIR) (TR = 6.8 ms, TE = 3.2 ms, TI = 1650 ms, matrix = 256 × 207, FOV = 250 × 250 × 184 mm^3^, slice thickness = 1.2 mm), and DKI (TR = 3900 ms, TE = 810 ms, matrix = 80 × 80, FOV = 230 × 90 × 230 mm^3^, slice thickness = 3 mm). The DKI was collected using a single-shot, spin echo-echo planar imaging sequence, with two non-zero b values (b = 1000 and 2000 s/mm^2^) applied along fifteen gradient encoding directions separately in addition to the b = 0 s/mm^2^ image for each subject. 

### 2.3. ^18^F-Flutemetamol PET Acquisition

All the participants had to fast for at least six hours before the PET acquisition. A bolus of ^18^F-flutemetamol (about 5 mCi) was injected intravenously for each patient. The PET scanning started after around 1.5 h of injection using an integrated PET-computed tomography (PET-CT) scanner with a 3D list mode. The duration of scanning time was 30 min. Filtered back-projection reconstruction was adopted with a slice thickness of 2 to 4 mm, and matrix size was 128 × 128 with a pixel size of 2 mm. Details of the acquisition scheme have been reported previously [27].

### 2.4. Volume Analysis

**Figure 1:**
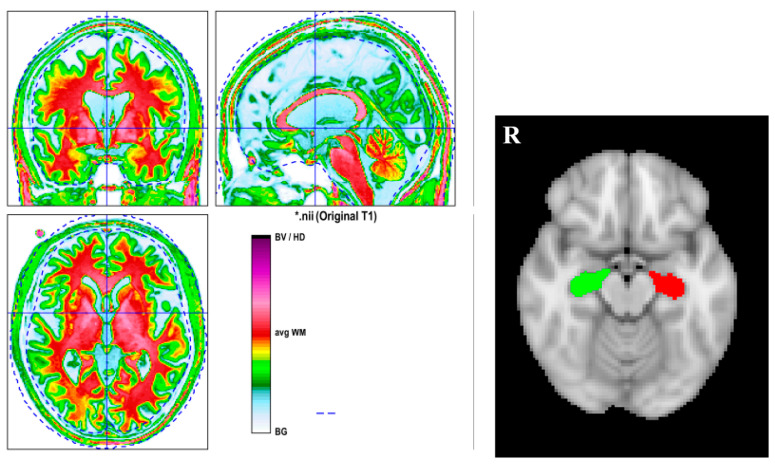
The left side figure is the segmentation result of a subject. The right side figure is the hippocampal ROI from CAT12. The computational anatomy toolbox, version 12.6 (CAT12), was applied to 3D T1-MPRAGE images to perform the brain segmentation. BG = background, CSF = cerebrospinal fluid, CGM = cortical GM, GM = grey matter, WM = white matter. The red color indicates WM; the green color denotes GM; and the yellow color represents the interface between WM and GM. * just represents the name of the subjects.

Reorigin was first conducted for the 3D T1-MPRAGE to make the following segmentation much more accurate with statistical parametric mapping, version 12 (SPM12), running on MATLAB R2020, version 9.8.0 (The MathWorks, Natick, MA, USA). Then, computational anatomy toolbox, version 12.6 (CAT12), was applied to the 3D T1-MPRAGE images to perform the brain segmentation for everyone separately. The segmentation quality was checked visually one by one. Figure 1 displays the segmentation result of one subject. Brain volumes (GM, WM, and total intracranial volume (TIV)) can be obtained automatically. By using the function “extracting ROI data” in CAT12, the volume of the bilateral hippocampus for each participant can be obtained. 

### 2.5. DKI Analysis

MRIcroGL (https://www.nitrc.org/projects/mricrogl, accessed on 20 June 2022) was applied to transfer the images from DICOM to NIfTI format for each subject. Firstly, image quality was checked visually one by one. Then FMRIB software library (FSL) (https://fsl.fmrib.ox.ac.uk/fsl/fslwiki/, accessed on 20 June 2022) [33], version 6.0, was used to perform the eddy current and motion correction for the diffusion images. Gradient tables of b values were also rotated based on the transformation derived from the last step [34]. Then the nonbrain tissues were removed to make the following registration and normalization much more rapid and accurate. After these preprocessing steps, the toolbox diffusion kurtosis estimator (DKE) was implemented to derive the DKI measurements (FA, MD, AxD, RaD, and MK) [35]. 

After the abovementioned preprocessing steps, the two metrics maps, MD and MK, were registered into the Montreal Neurological Institute’s (MNI) standard space through the following two-way registration method (we only focused on the MD and MK of bilateral hippocampus considering that it is hard to interpret the alterations of FA, AxD, and RaD in GM, especially AxD and RaD, which are often used to assess the microstructural abnormalities of WM). Firstly, the b0 image was linearly registered into the native 3D T1-MPRAGE space using the function “flirt” [36] and implemented in FSL; secondly, the native 3D T1-MPRAGE image was nonlinearly normalized into standard MNI T1 image using “fnirt” [37], a function of FSL; and, thirdly, the transformations derived from these two steps were applied into MD and MK maps in the native diffusion space and transferred into standard MNI space using the function “applywarp” in FSL. The normalization quality was checked individually. 

**Figure 2:**
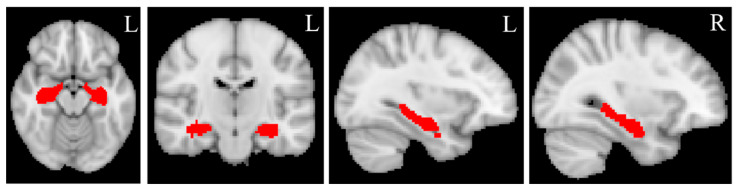
Hippocampal masks. The bilateral hippocampal masks are created from Automated anatomical labeling (AAL) atlas, version 1, in standard MNI space using “fslmaths”, a function implemented in FSL. L = left, R = right. The red color represents the bilateral hippocampal masks.

**Figure 3:**
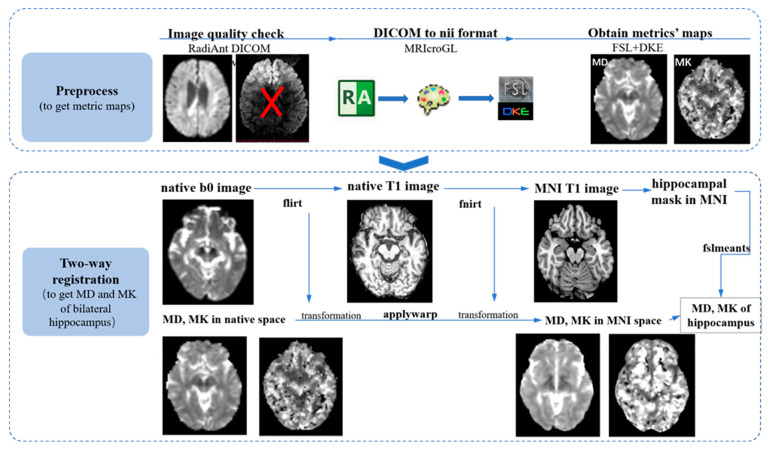
The flowchart of imaging processing. The image processing pipelines are mainly divided into three steps: preprocessing step (this mainly includes visual image quality checking using the software RadiAnt DICOM Viewer (https://www.radiantviewer.com/, accessed on 20 April 2022), converting the image format from DICOM into NIFIT using MRIcroGL; obtaining measurement maps using FSL and DKE together); and two-way registration (MD and MK maps were normalized into standard space using FSL based on Linux system. Firstly, the b0 image was registered into native T1 space using “flirt”; secondly, native T1 was normalized into MNI T1 space using “fnirt”; thirdly, the transformations from the last two steps were applied to native MD/MK and aligned into standard space and extraction of values using “fslmeants”.

The binary masks of the bilateral hippocampus (Figure 2) were created from the automated anatomical labelling (AAL) atlas (https://www.gin.cnrs.fr/en/tools/aal/, accessed on 20 June 2022) [38], version 1, in the MNI space using “fslmath”, a function of FSL. MD and MK values for the bilateral hippocampus were extracted from the MD and MK maps in the standard space using the function “fslmeants” in FSL for each participant. Figure 3 displays the whole processing steps of DKI.

### 2.6. ^18^F-Flutemetamol PET Analysis

The ^18^F-Flutemetamol PET images were evaluated as amyloid positive or negative by a neuroradiologist who had completed the training program developed by GE Healthcare for the interpretation of ^18^F-Flutemetamol images [39].

### 2.7. The Definition of Total Hippocampal Measurements

MD and MK were calculated for each participant. Nevertheless, the total and bilateral hippocampus measurements were included in the analysis. The total hippocampal volume was defined as total hippocampal volume = left hippocampal volume + right hippocampal volume. The total MD was defined as total MD = (left hippocampal MD + right hippocampal MD)/2. The total MK was defined as total MK = (left hippocampal MK + right hippocampal MK)/2.

### 2.8. The Definition of Percentage Change

The percentage change for each disease group compared to NCs was defined as the ratio between the absolute difference and the value of NCs. For example, in the pair-wise comparison of AD and NCs, the percentage change of right hippocampal MK for AD was defined as the ratio between their absolute MK difference (right hippocampal MK difference between AD and NCs) and the MK of NCs. Similarly, in the comparison between AD and MCI, the percentage change of right hippocampal MK for AD was calculated as the ratio between their absolute difference (the difference of right hippocampal MK between the AD and MCI groups) and the MK value of MCI.

### 2.9. Statistical Analysis

All statistical analyses were performed using IBM SPSS statistics version 27 (IBM, Chicago, IL, USA, https://www.ibm.com/products/spss-statistics, accessed on 22 June 2022). Results were regarded as significant when the *p*-value was smaller than 0.05. GraphPad Prism (version 8.0.0 for Windows, GraphPad Software, San Diego, California, USA) was used to plot statistical figures.

The Shapiro–Wilk test was applied to check the normality of continuous variables (age, HK-MoCA, brain volume, MD, and MK). Analysis of variance (ANOVA) was adopted to test the differences in age and HK-MoCA, and the chi-square test was applied to detect the difference in gender among these four groups.

For comparison of volume, MD and MK of the bilateral hippocampus among these four groups (NC, SCD, MCI, and AD) and analysis of covariance (ANCOVA) followed by Bonferroni post-hoc analysis for multiple comparisons correction was used, adjusting for age and gender.

For comparing the diagnosis performance of volume, MD, and MK of the bilateral hippocampus, the receiver operating characteristic (ROC) curve was plotted for every measurement (i.e., volume, MD, and MK) for each pair-wise comparison. In addition, the area under curve (AUC) values were also computed for each measurement.

## 3. Results

### 3.1. Participant Characteristics

The demographic and clinical data are provided in Table 1. There was no significant difference in age among all the groups. HK-MoCA showed a significant difference in the post-hoc pair-wise comparison between AD vs. MCI, and MCI vs. SCD. Sex also did not show significant differences among all these subgroups. 

### 3.2. AD vs. NC

**Figure 4:**
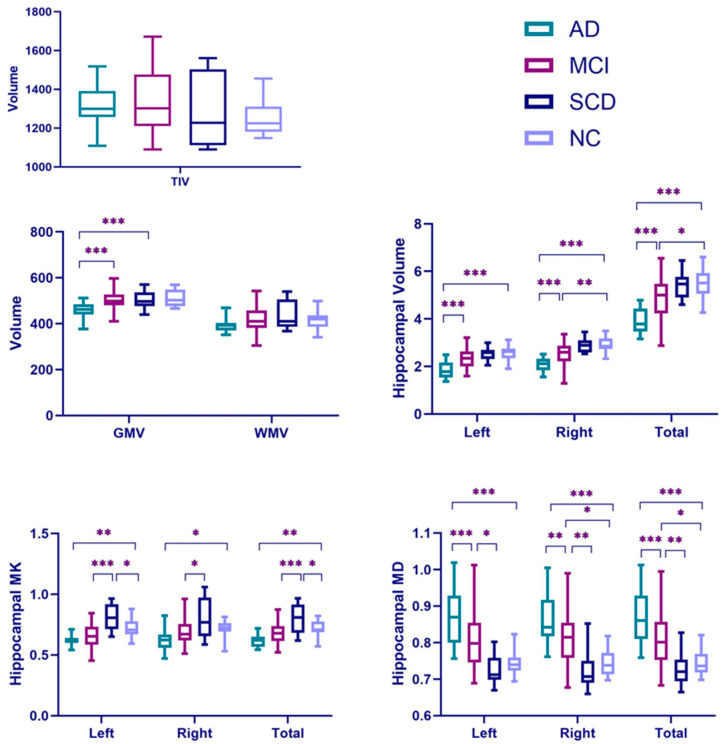
Boxplots of brain volume, hippocampal volume, MD, and MK. Total Hippocampal volume = left hippocampus volume + right hippocampus volume; total hippocampal MD = (left hippocampus MD + right hippocampus MD)/2; total hippocampal MK = (left hippocampus MK + right hippocampus MK)/2. GMV = grey matter volume; WMV = white volume; TIV = total intracranial volume; MK = mean kurtosis; and MD = mean diffusivity. * *p* ≤ 0.05, ** *p* ≤ 0.01, *** *p* ≤ 0.001.

**Figure 5:**
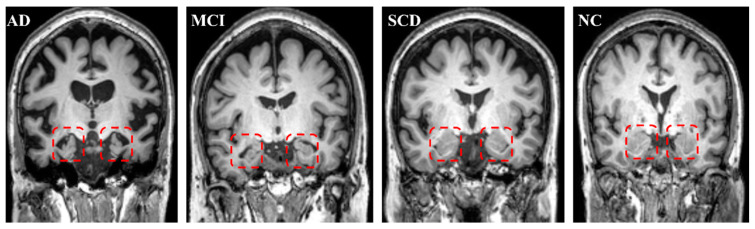
Typical subjects for AD, MCI, SCD, and NCs. The red square contains the bilateral hippocampus. The atrophy of the bilateral hippocampus for AD is the most severe; MCI is intermediate. There was no hippocampal atrophy for SCD compared with NCs.

**Figure 6:**
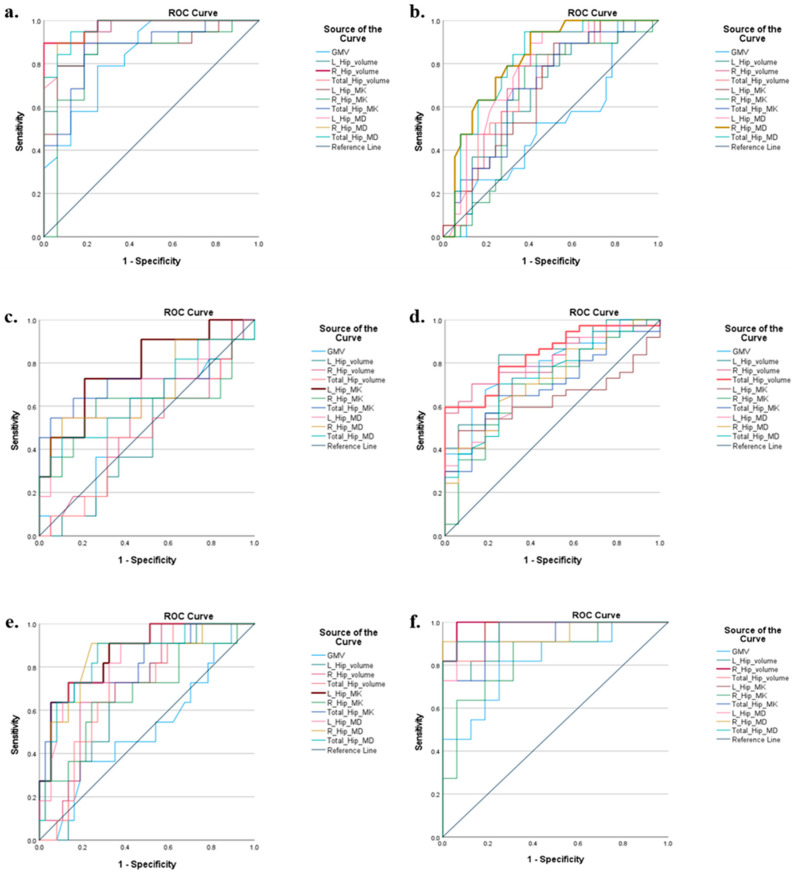
ROC curve for each pair-wise comparison. The statistical software SPSS (version 27) was used to do the ROC analysis. Total_Hip_MD = (left hippocampus MD + right hippocampus MD)/2; Total_Hip_MK = (left hippocampus MK + right hippocampus MK)/2; Total_Hip_volume = left hippocampus volume + right hippocampus volume. AD = Alzheimer’s disease; MCI = mile cognitive impairment; SCD = subjective cognitive decline; NC = normal control; (**a**) AD vs. NC; (**b**) MCI vs. NC; (**c**) SCD vs. NC; (**d**) AD vs. MCI; (**e**) MCI vs. SCD; and (**f**) AD vs. SCD.

AD showed a significant, whole GM volume (GMV) reduction compared with NCs (*p* < 0.001). For the WM volume (WMV) and total intracranial volume (TIV), AD did not display a significant decrease compared with NCs (Table 2). However, volume measurements showed a significant decline in the AD group on both the left and right hippocampus compared with those in the NC group (*p* < 0.001 for both sides). The total hippocampus volume also significantly decreased compared with NCs (Table 2 and Figure 4). Figure 5 displays the bilateral hippocampal volume of typical AD, MCI, SCD, and NC subjects. AD showed the most apparent bilateral hippocampal volume reduction. AD also exhibited a significant decrease in the bilateral hippocampus MK and an increase in MD compared with those in NC, respectively (Table 2 and Figure 4). The right hippocampus volume showed a pronounced percentage change in the AD group (Table 3). In the ROC analysis, the right hippocampal volume showed the most prominent area under the curve value (AUC = 0.977) (Table 4 and Figure 6).

### 3.3. MCI vs. NC

MCI displayed significant volume reduction in the right and total hippocampus compared with NCs. It also showed a significant increase in MD in the right and total hippocampus compared with NCs (*p* = 0.011 and *p* = 0.017). For other variables, such as GMV, WMV, TIV, and hippocampal MK, no significant changes were observed in the MCI group compared to NCs (Table 2). Right hippocampal volume showed the most significant percentage change compared with NCs (Table 3). In the ROC analysis, the MD of the right hippocampus was the most sensitive discriminator for MCI (AUC = 0.819) (Table 4 and Figure 6).

### 3.4. SCD vs. NC

Only the left and the total hippocampal MK of the hippocampus showed significant differences compared with NCs for SCD. Meanwhile, the left hippocampal MK also displayed the biggest significant percentage change compared with NCs among all the measurements, and it was also the most sensitive biomarker for SCD diagnosis (AUC = 0.775) (Table 4).

### 3.5. AD vs. MCI

AD showed a noticeable reduction in GMV and bilateral hippocampal volume compared with MCI (*p*-value was smaller than 0.001 for all pair-wise analyses) (Table 2 and Figure 4). For the MD measurement, AD displayed a significant increase compared with MCI (*p* < 0.001 for the left hippocampus, *p* = 0.002 for the right hippocampus, and *p* < 0.001 for the total MD). Left hippocampal volume showed the most significant percentage change for AD compared with MCI (Table 3). In the ROC analysis, the total hippocampal volume had the best potential to discriminate AD from MCI (AUC = 0.836) (Table 4).

### 3.6. MCI vs. SCD

No volume variables showed significant differences in the pair-wise comparison between MCI and SCD (Table 2). But the microstructural variables MD and MK showed significant differences (Table 2). MK decreased and MD increased in the bilateral hippocampus for MCI compared with SCD (Table 2). The left hippocampal MK showed the most significant difference between MCI and SCD, which was the best discriminator (AUC = 0.865).

### 3.7. AD vs. SCD

AD displayed significant differences compared to SCD for all the variables (Table 2). The right hippocampal volume exhibited the most prominent AUC value (AUC = 0.977) (Table 4 and Figure 6)

## 4. Discussion

This cross-sectional study showed the diagnostic values of hippocampal volume, MD, and MK in different AD stages. DKI might be a sensitive marker for early AD detection in the SCD stage. Interestingly, our primary finding was that the microstructural impairment in the AD spectrum was not linear but curvilinear. Compared to NCs, the MK of the left hippocampus was increased for SCD while it was decreased for AD. As expected, the AD group’s hippocampal volume change was more pronounced than MCI and NCs. In contrast, the left hippocampal MK change was superior to the volume change in the SCD group, suggesting that it might serve as a marker for early diagnosis of SCD. In line with this observation, the left hippocampal MK showed the most significant change in MCI compared with SCD. Therefore, hippocampal volume (macrostructural changes) was the best discriminator for diagnosing the dementia stage of AD. In contrast, hippocampal MD or MK (reflecting microstructural changes) was the best indicator for the predementia stage of AD (e.g., right hippocampal MD for MCI and left hippocampal MK for SCD). In discriminating AD from MCI, the total hippocampal volume was the best predictor. In differentiating MCI from SCD, hippocampal MK (left) was the best discriminator.

These results suggest that in the predementia SCD and MCI stages, microstructural changes probed by MD and MK may be much more apparent. In contrast, in the later stage of AD (i.e., the dementia stage), macrostructural alterations (i.e., volume changes) may be a superior indicator. This observation was in line with previous results by Nan-Jie Gong et al. [22].

**Figure 7:**
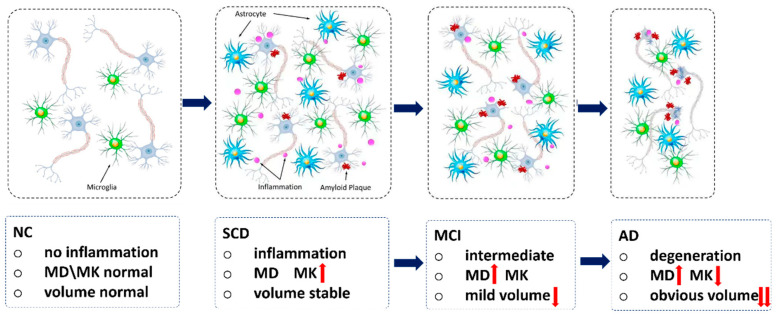
The assumptions of pathological changes in AD spectrum. In SCD stage, the amyloid plaque and other risk factors might trigger the inflammatory process associated with increased microglia and astrocytes. These pathological processes eventually lead to increased cellular density and microenvironment complexity in hippocampal tissues, so the MK would increase. In MCI stage, the expanded extracellular space (due to the axonal and neuronal loss) outweighed the inflammation, so the water diffusion was not hindered but increased, and MD was increased. In AD stage, brain atrophy (neurodegeneration or neuronal body loss) will be predominant. AD = Alzheimer’s disease; SCD = subjective cognitive decline. We plotted this figure by ourselves. The ascending red arrow represents increase; the descending red arrow represents decline.

Our main observation was the curvilinear change of MK along the disease progression from SCD to AD. The pathological explanations are provided below (Figure 7). In the preclinical SCD stage, microstructural changes are predominant, owing to the inflammation triggered by slight amyloid deposition [40], where inflammation has a protective function. The amyloid plaque and other risk factors might trigger the inflammatory process associated with increased microglia and astrocytes which secrete inflammatory mediators such as proinflammatory cytokines and chemokines [16], which were also involved in the onset of another common type of neurodegenerative disorder, Parkinson’s disease [41]. The specific pathogenesis of the two common neurodegenerative diseases may be different, but they may share common pathways, such as inflammation. These pathological processes eventually lead to increased cellular density and microenvironment complexity in hippocampal tissues (Figure 7). This hypothesis may help explain why our findings showed that the MK of left hippocampus was increased in the SCD stage. A previous preclinical study [42] by Jelle Praet et al. showed MK also increased in the cingulate cortex of APP/PS1 transgenic mice compared to wild-type (WT) mice, owing to the amyloid deposition. This suggested that increased cellular microstructural/microenvironment complexity could lead to the observable changes in DKI, which might support our findings and hypothesis. In addition, MK is the only measurement that showed significant changes in the SCD group, suggesting that DKI may be a useful biomarker to detect AD at an early stage. This was also consistent with previous animal studies [42,43].

In the prodromal MCI stage, insignificant volume changes were detected in the left hippocampus, while discrepancies were noticed in the right hippocampus. This result slightly deviated from the results in a previous study [44]. The bilateral differences in hippocampal volume reductions might be due to the fact that the degeneration of the bilateral hippocampus is not simultaneous [44]. In the MCI stage, water diffusivity of the right hippocampus was also slightly increased compared to NCs. At the MCI stage, the expanded extracellular space (due to the axonal and neuronal loss) outweighed the inflammation [45], so the water diffusion was not hindered but increased compared to SCD. The result of a previous study supported our observation [46], although that study didn’t differentiate diffusion measurements in the bilateral hippocampus. Other studies also observed the increased hippocampal MD in MCI compared to NC [24,25,47].

Our results showed that hippocampal atrophy became predominant in the AD stage, where volume loss is much more evident than the microstructural changes [19,48]. We observed that right not left hippocampal volume loss was the best discriminator for diagnosing AD. The asynchronous neurodegeneration and atrophy progression of the bilateral hippocampus might account for this [15]. Further studies on a larger patient population might be needed to confirm such bilateral changes of the hippocampus in different patient groups.

Our current study is limited by the small sample size and the use of 15 diffusion directions in DKI measurements. Meanwhile, we still observed significant changes in volumetry and DKI measurements at different stages of AD; therefore, we believe that the relatively small sample size did not affect the main conclusion of this study. Fifteen diffusion directions were used in the DKI due to the limited scan time for each patient. Previous studies showed that 15 directions still can characterize the diffusion and kurtosis of the tissues according to the definition of the DKI model, non-Gaussian along the b-value axis [12,13]. It was sufficient to detect significant differences in MD and MK for each disease group compared to NCs. Furthermore, prolonging the scan time for more diffusion direction is not desirable in clinical situations. Therefore, we used 15 diffusion directions, which could not affect the conclusion of this study.

## 5. Conclusions

This paper adds new neuroimaging tools in the differential diagnosis of the AD spectrum using the DKI technique. In the dementia stage (AD), the best discriminator is the macrostructural index (right hippocampal volume); in the predementia stage (preclinical stage SCD and prodromal stage MCI), the best indicators are microstructural measurements (left hippocampal MK for SCD and right hippocampal MD for MCI). This paper also adds new ways to ascertain early stage SCD using DKI.

## Figures and Tables

**Table 1:** Demographic and clinical data.

	Different Disease Group				*p*-Value				
	NCs	SCD	MCI	AD	SCD vs. NC	MCI vs. NC	AD vs. NC	AD vs. MCI	MCI vs. SCD
Age (year)	73.1 ± 5.9	71.0 ± 7.2	75.4 ± 7.4	75.0 ± 8.1	0.477	0.305	0.462	0.843	0.080
Sex (F:M)	15:4	7:4	21:16	11:5	0.169	0.096	0.233	0.357	0.621
HK-MoCA	N/A	28.1 ± 2.1	20.9 ± 4.7	12.5 ± 5.8	N/A	N/A	N/A	<0.01	<0.01

Note: Age and HK-MoCA are presented as mean ± standard deviation. Analysis of variance (ANOVA) was used to detect the differences in age and HK-MoCA among all the groups; Chi-square test was used to detect the differences in sex among all groups. NC = normal control, SCD = subjective cognitive impairment, MCI = mild cognitive impairment, AD = Alzheimer’s disease, HK-MoCA = Hong Kong version of Montreal cognitive assessment. F = female, M = male. N/A = not applicable.

**Table 2:** Brain volume, hippocampal volume, MD and MK of AD spectrum and NC.

	AD	MCI	SCD	NC	*p* Value	Pair-Wise *p* Value					
						AD vs. NC	MCI vs. NC	SCD vs. NC	AD vs. MCI	MCI vs. SCD	AD vs. SCD
Brian Volume											
GMV	455.44 ± 41.60	502.49 ± 42.99	505.45 ± 37.68	507.74 ± 35.24	<0.001	<0.001	n/s	n/s	<0.001	n/s	0.011
WMV	392.63 ± 30.08	417.08 ± 52.85	439.45 ± 59.83	416.42 ± 37.49	n/s	n/s	n/s	n/s	n/s	n/s	n/s
TIV	1312.4 ± 108.9	1329.8 ± 152.9	1299.5 ± 185.47	1249.21 ± 85.7	n/s	n/s	n/s	n/s	n/s	n/s	n/s
Hippocampus Volume											
Left	1.829 ± 0.343	2.336 ± 0.446	2.543 ± 0.272	2.551 ± 0.330	<0.001	<0.001	0.099	n/s	<0.001	n/s	<0.001
Right	2.076 ± 0.290	2.571 ± 0.476	2.901 ± 0.297	2.904 ± 0.304	<0.001	<0.001	0.008	n/s	<0.001	n/s	<0.001
Total	3.905 ± 0.575	4.907 ± 0.896	5.443 ± 0.553	5.456 ± 0.626	<0.001	<0.001	0.021	n/s	<0.001	n/s	<0.001
Hippocampus MK											
Left	0.6222 ± 0.0408	0.6616 ± 0.0926	0.8109 ± 0.0985	0.7200 ± 0.0773	<0.001	0.008	n/s	0.032	n/s	<0.001	<0.001
Right	0.6205 ± 0.0871	0.6857 ± 0.0932	0.7932 ± 0.1593	0.7173 ± 0.0670	<0.001	0.050	n/s	n/s	n/s	0.040	<0.001
Total	0.6214 ± 0.0546	0.6737 ± 0.0819	0.8020 ± 0.1106	0.7186 ± 0.0627	<0.001	0.005	n/s	0.046	n/s	<0.001	<0.001
Hippocampus MD											
Left	0.8729 ± 0.0768	0.8027 ± 0.0706	0.7247 ± 0.0392	0.7435 ± 0.0319	<0.001	<0.001	0.054	n/s	<0.001	0.019	<0.001
Right	0.8701 ± 0.0744	0.8119 ± 0.0709	0.7260 ± 0.0514	0.7417 ± 0.0352	<0.001	<0.001	0.011	n/s	0.002	0.007	<0.001
Total	0.8715 ± 0.0720	0.8073 ± 0.0685	0.7254 ± 0.0443	0.7426 ± 0.0331	<0.001	<0.001	0.017	n/s	<0.001	0.008	<0.001

Note: The measurements values are expressed as mean ± standard deviation; n/s = no significant; GMV = grey matter volume, WMV = white matter volume, TIV = total intracranial volume; MK = mean kurtosis; MD = mean diffusivity. Total hippocampal volume = left hippocampus volume + right hippocampus volume; total hippocampal MD = (left hippocampus MD + right hippocampus MD)/2; and total hippocampal MK = (left hippocampus MK + right hippocampus MK)/2.

**Table 3:** Percentage change of measurements for each pair-wise comparison.

	GMV	Hip Volume (%)			Hip MK (%)			Hip MD (%)		
		Left	Right	Total	Left	Right	Total	Left	Right	Total
AD vs. NC	−10.3	−28.2	−28.5	−28.4	−13.6	−13.5	−13.4	17.4	17.3	17.4
MCI vs. NC	−1.0	−8.4	−11.5	−10.1	−8.1	−4.4	−6.3	8.0	9.5	8.7
SCD vs. NC	−0.5	0.3	0.1	0.2	12.6	10.6	11.6	−2.5	−2.1	−2.3
AD vs. MCI	−9.4	−21.7	−19.3	−20.4	−5.9	−9.5	−7.8	8.7	7.2	7.9
MCI vs. SCD	−0.6	−8.1	−11.4	−10.6	−18.4	−13.5	−16.0	10.8	11.8	11.3
AD vs. SCD	−9.8	−28.1	−28.4	−28.3	−23.3	−21.7	−22.5	20.4	19.8	20.1

Note: The percentage change for each disease group compared to NC was defined as the ratio between the absolute difference and the value of NC. For example, in the comparison between AD and NC, the percentage change of right hippocampal MK for AD was defined as ratio between their absolute MK difference (right hippocampal MK difference between AD and NC) and the MK of NC. Total hippocampal volume = left hippocampus volume + right hippocampus volume; total hippocampal MD = (left hippocampus MD + right hippocampus MD)/2; total hippocampal MK = (left hippocampus MK + right hippocampus MK)/2; GMV = grey matter volume; MD = mean diffusivity; and MK = mean kurtosis.

**Table 4:** AUC values for each pair-wise differential diagnosis.

	AD vs. NC	MCI vs.NC	SCD vs. NC	AD vs. MCI	MCI vs. SCD	AD vs. SCD
GMV	0.844	0.507	0.505	0.791	0.514	0.818
Hippocampus Volume						
Left	0.934	0.661	0.526	0.814	0.666	0.943
right	0.977	0.718	0.505	0.821	0.727	0.986
total	0.974	0.701	0.502	0.836	0.700	0.966
Hippocampus MK						
Left	0.882	0.676	0.775	0.628	0.865	0.977
Right	0.829	0.647	0.608	0.698	0.686	0.841
Total	0.865	0.686	0.722	0.691	0.821	0.920
Hippocampus MD						
Left	0.962	0.770	0.660	0.749	0.844	0.966
Right	0.967	0.819	0.675	0.717	0.843	0.949
Total	0.967	0.807	0.651	0.735	0.848	0.972

Note: Total hippocampal volume = left hippocampus volume + right hippocampus volume; total hippocampal MD = (left hippocampus MD + right hippocampus MD)/2; total hippocampal MK = (left hippocampus MK + right hippocampus MK)/2; GMV = grey matter volume; MD = mean diffusivity; MK = mean kurtosis; AUC = Area under curve. The biggest AUC value for each pair-wise comparison is expressed in bold.

## Data Availability

The clinical data and MRI images are not publicly available for patient privacy protection purposes.
